# Review of Current Strategies to Address Micronutrient Deficiencies (MNDs) in Ghana: A Scoping Review

**DOI:** 10.1155/jnme/6652716

**Published:** 2025-01-29

**Authors:** Jolene Mateko Azagba-Nyako, Charles Tortoe, Paa Toah Akonor, Alice Padi, Jackline Boateng, Richard Otwey

**Affiliations:** ^1^Food Chemistry and Nutrition Division, Council for Scientific and Industrial Research-Food Research Institute, P.O. Box M20, Accra, Ghana; ^2^Food Technology Research Division, Council for Scientific and Industrial Research-Food Research Institute, P.O. Box M20, Accra, Ghana; ^3^Food Microbiology and Mushroom Research Division, Council for Scientific and Industrial Research-Food Research Institute, P.O. Box M20, Accra, Ghana

**Keywords:** Ghana, micronutrients deficiencies, MNDs, strategies

## Abstract

To achieve the Sustainable Development Goals by 2030, low-income countries like Ghana will require strategizing towards sustainable reduction in micronutrient deficiency (MND) diseases and MND-related morbidity and mortality. A scoping review was conducted to identify the policy framework around MND intervention, the actors implementing MND-related strategies and documented evidence on which strategies worked across implementation stakeholders. Forty-six peer-reviewed articles were selected (*n* = 46) including studies on nutrition-sensitive intervention studies (*n* = 15) and nutrition-specific intervention (*n* = 31). Eligibility criteria for inclusion of information from articles and publications were based on report findings on nutrition-related programmes and policies conducted and implemented in Ghana and should have been published between 2000 and 2024. Databases adopted for this scoping review include Google Scholar, PubMed, ScienceDirect, AGORA, Hinari and JSTOR. This scoping review obtained a comprehensive picture of the nutrition situation in Ghana by gathering nutrition surveillance data depicting the different strategies employed to combat MNDs in Ghana. The scoping review identified several MND intervention strategies; however, the sustainability of the strategies required effective implementation guidelines, policies and programmes that address dietary deficits specific to a particular population.

## 1. Introduction

Micronutrient deficiencies (MNDs) are significant contributors to childhood morbidity and mortality across the globe with the highest disease burden affecting the poorest populations in the global south mainly Asia and Africa [1]. In 2020, close to half of all deaths in children under 5 could be attributed to some sort of MNDs. MNDs increase the child's risk of being infection-prone and reduce the body's ability to fight off the infection and even delay recovery [2, 3]. Projections for the next decade suggest a worsening downward trend of the burden of disease attributable to MNDs [4].

From the Ghanaian perspective, despite recent improvements in Ghana's health sector, MNDs such as vitamin A, iron, zinc and iodine deficiencies and their associated comorbidities are among the highest contributors to disease burden in Ghana. The prevalence of anaemia among pregnant women during their first three months of gestation was averaged to be 58% among pregnant Ghanaian women in a longitudinal study conducted by [5], while anaemia among nonpregnant women was reported to be 22% from a 2017 national survey [6]. Wegmüller, Bentil and Wirth [7] reported that 21.7% of reproductive women in the northern belt of Ghana were anaemic, whereas nationally, 35.6%, 21.5% and 20.8% of preschool children are considered anaemic, iron deficient and vitamin A deficient, respectively. The dataset [8] showed that nearly 35% of all children under 5 years were affected by anaemia and 25% were vitamin A deficient. Among women of reproductive age over 7% were vitamin B_12_ deficient and nearly 22% folate deficient [9]. Additionally, the prevalence of overweight and obesity is progressively increasing, 23.4% of adults aged 25 years and over 40% of women living with some form of adiposity [9].

The increased availability of high-calorie foods such as rice, sugar and tubers altered dietary patterns and led to excessive energy intake, low dietary diversity and overweight and obesity [10]. On the other hand, one of the many hindrances to the consumption of nutritious food is the seasonal availability of fruits and vegetables and the high cost of animal products; however, interventions along the value chain of supply and demand are likely to improve accessibility and affordability of nutritious foods [11]. The complications of MNDs are many. Some of the adverse effects of MNDs include complications associated with childbirth, impaired immune system, restrictions to children's growth and development, and aggravating chronic illness which results in lowering the potentials of populations worldwide [12, 13]. There is a growing concern that if these trends are allowed to continue without mitigation, Ghana will soon be facing a crippling triple burden of malnutrition (underweight, overweight and hidden hunger in the same population).

National MND mitigating efforts have been stalled by the lack of continuous plausible nutrition surveillance data on one hand and poor impact analysis on the other. This makes it difficult to identify and quantify the full extent of the magnitude of the prevalence of MNDs in Ghana, stalls the harmonization of national efforts and prevents the dissemination of the current best practices in terms of strategies for MNDS prevention.

This review sought to present a comprehensive picture of MND strategies that have been utilized in Ghana over the last 2 decades by gathering and reviewing data about the different strategies employed to combat MNDs in Ghana, and this review identified and mapped out and examined strategies available for reducing MNDs considering target population, input, potential for integration with other interventions, sustainability and key findings. The review discusses the different forms of strategies depending on efficacy, effectiveness, potential adverse effects, constraints to implement and explored opportunities to optimize these strategies to promote sustainable impact.

## 2. Research Methodology

### 2.1. Approach

The review was preceded by a mapping out of key national nutrition-related legal frameworks and policies implemented by key agencies with detailed attention to policies relevant to addressing MNDs in Ghana. Some of these key national policies include the following:

• National plan of action: Food and Nutrition 2000
• National Health Policy: Creating Wealth through Health 2015
• Ghana National Nutrition Policy 2014–2017

**Figure 1:**
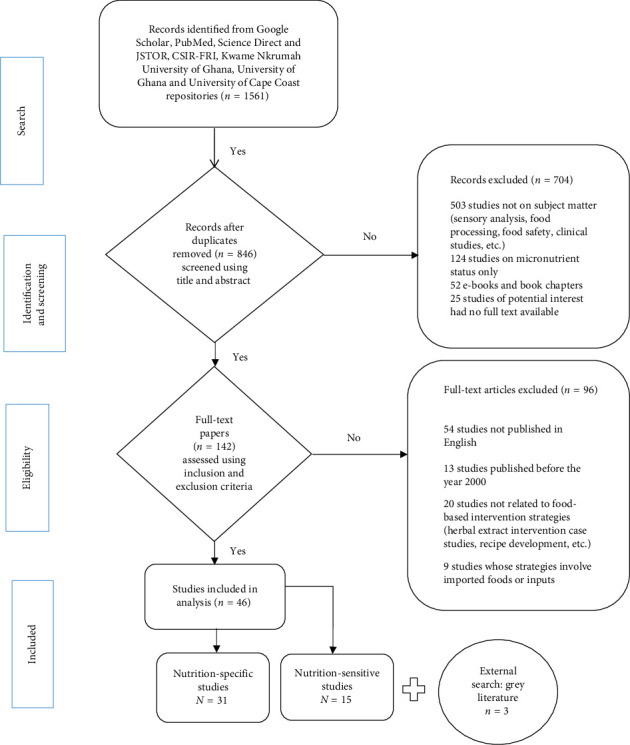
Data search strategy showing search term.

The review approach shown in Figure 1 aligns with the Preferred Reporting Items for Systematic Reviews and Meta-Analyses Extension for Scoping Reviews guidelines. The scoping questions that aided the search, selection and screening process and mapping of data were based on an adaptation of the framework outlined by Arksey and O'Malley [14] and Colquhoun et al. [15] as shown in Table 1. The synthesis of existing knowledge, identification of key concepts and evidence, and possible gaps in research were conducted using a series of exploratory research questions listed in Table 2.

### 2.2. Search Strategy

The search for records was performed using Google Scholar, PubMed, Science Direct, AGORA, Hinari and JSTOR databases as well as Ghanaian University databases and repositories including the CSIR-FRI repository, Kwame Nkrumah University of Ghana, University of Ghana and University of Cape Coast.

The search considered records of studies that included terms relating to phrases Micronutrients Deficiencies (MNDs) AND Ghana, National Micronutrients Deficiencies SURVEY and nutrition interventions and nutrition programming in Ghana. Keywords used included; Food-based interventions AND Nutrition interventions AND Ghana, nutrition-specific interventions in Ghana, Fortification, dietary diversification AND supplementation interventions in Ghana, and nutrition-sensitive interventions in Ghana. Subsequent keyword searches were used to optimize the search “nutrition programing” AND “micronutrient deficiency in Ghana” OR “nutrition intervention: f∗” OR “nutrition policy” OR “malnutrition prevention AND Ghana” OR “nutrition improvement∗” OR “fortification∗” OR “supplementation” OR “complementary feeding” OR “dietary diversity “OR “crop varieties” OR “food-based approach∗” OR “indigenous food” OR “nutrition sensitive” OR “nutrition and agriculture∗” OR “improved variety∗” OR food biodiversity∗” OR “nutrition∗ education.” Records on the strategies to address MNDs in Ghana were collected from open access papers, thesis and reports of studies conducted in Ghana or of relevance to Ghana within two search windows: 07/09/2017–2/04-2018 and an updated search between 13/11/2022 and 18/02/2023.

### 2.3. Inclusion and Exclusion Criteria

In selecting eligible articles with topics related to MNDs in Ghana (both quantitative and qualitative research), articles fulfilling the following criteria were identified for review:

• Written in English
• Published between 2000 and 2024
• Available online/open access
• Relevance to food-based strategies (fortification, supplementation, dietary diversity)
• Relevance to food and nutrition security with special emphasis on integrated strategies, agri-food–nutrition–health linkages

Specific exclusion criteria were applied in the full-text review:

• Studies promoting the use of nonindigenous food and imported foods
• Sensory analysis study data were excluded.

Search results were transferred to an MS Excel spreadsheet, and duplicates were removed. The search retrieved 1056 unique articles as shown in Figure 1. The two rounds of screening of titles and abstracts where searches were performed to identify articles that met the open access, and in the process, 704 studies were excluded: 503 irrelevant records, including those off the topic of interest, 124 records of studies focus on micronutrient status only, 52 e-books, books and book chapters. Also 25 potential abstracts were online but excluded due to the unavailability of the full text. In the secondary screening for eligibility, 109 records were selected for full-text screening using the inclusion and exclusion criteria. The initial screening was done independently by JMAN and followed up with an updated screening by two authors (Jackline Boateng and Richard Otwey) who agreed on the selection of 46 peer-reviewed articles. Each record was charted by the extraction of details on the research name, target population, research design, sample size, geographic location methods, key indicators, findings, authors and the date on an MS Excel spreadsheet.

## 3. Results and Discussions

### 3.1. Description of Results

In terms of study design, fourteen were largely randomized controlled trials where studies compared interventions with a control group and four national mandatory fortification programmes, whereas thirty were observational studies that reported outcomes linked to intake of either formulated food, fortified foods or a diverse diet and one study was quasiexperimental. Ten nutrition-sensitive strategies in agriculture focused on interventions to diversify diets by increasing access to a variety of food groups, increasing household food production, agency and access to nutritious food, and utilization and consumption of micronutrient-reach foods. The other five nutrition-sensitive studies focused on nutrition education while the remaining focused on behaviour change communication on water and sanitation and hygiene nutrition education with women, infants and young children and school-aged children being the main target beneficiaries of intervention strategies.

### 3.2. Policies and Implementers

#### 3.2.1. What Are the Policies and Framework That Tackle MNDs?

As part of these efforts, the Ghana government renewed its mandate to focus on nutrition as a pivotal determinant of health and development. To this effect, several frameworks, policies and national strategies/agendas were put in place by the government of Ghana to ensure a consolidated effort from all relevant stakeholders to fill in the gaps in research and practice and to prevent duplication of nutritional efforts. The ‘Creating Wealth Through Health' policy was introduced to address the broader determinants of health such as agriculture, nutrition, physical activity and sanitation and paved the way for more integrated pathways that prioritize food security and nutrition as key components of healthcare delivery in Ghana. Healthcare delivery decision was no longer limited to the primary care centres but extended to tackling the socioeconomic root causes of ill health such as food insecurity, hygiene, nutrition, sanitation and physical well-being of vulnerable groups in the Ghanaian population. The ultimate aim was to promote pre-preventive health as a long-term strategy to reduce health sector expenditure, increase the productivity of the Ghanaian workforce and attain MDGS targets by 2015 [16].

The general approach of the national nutrition effort was to ensure the integrity of food nutrients from farm to fork. Hence, the government has made a substantial effort to prioritize nutrition in policy making and budget allocation and subsequently other food nutrition–centred policies, acts and laws.  Table 3 provides a summary of the government's nutrition policies that have been adopted over the years that helped Ghana to navigate the paths towards sustainable health. These policies together with other economic policies have championed a holistic approach to health by making food security, nutrition, potable water, environmental health, personal hygiene, regular physical activity and recreation key pillars crucial to achieving accelerated growth.

The Ministry of Health through its Nutrition Department is currently the main state agency with the mandate of budgetary provisions and expenditure for activities to address issues of malnutrition [38]. However, based on the government policy framework, the National Development Planning Commission (NDPC) is currently the government agency is responsible for the nutrition policy movement [28]. Considering the multifaceted nature of health strategy implementation, the Ghanaian government works together with partners and other stakeholders in health agriculture, nutrition health, research and academia to draft and implement policies, strategies and activities to help change the nutrition landscape of the country as well as aid Ghana attain the MDGs and subsequently the SDGs (Table 4). Due to the complexity of developing the Ghanaian nutrition landscape, national efforts have been buttressed by increased interest from civil society organizations such as the United Nations (UN), the Food and Agriculture Organization (FAO), the World Health Organization (WHO), European Union (EU) and many more who continue to champion nutrition advocacy, local capacity building, nutrition education efforts, disease control and WASH strategies (Table 5).

The multiagency efforts to promote the nutrition agenda subsequently resulted in Ghana making gradual but steady progress in reducing the prevalence of malnutrition in children under five years in the late 2000s [27]. Despite considerable improvements in the Ghanaian nutrition landscape, vitamin A, iron, zinc and iodine deficiencies and their associated comorbidities are among the severe public health concerns in Ghana. The trend lines for these MNDs seem to be worsening in certain demography. Similar estimates from a Ghana Health Services Survey dataset [8] indicated that 35% of all children under 5 years were anaemia deficient and 25% were vitamin A deficient. Among women of reproductive age, over 7% were vitamin B_12_ deficient, and nearly 22% were folate deficient [9]. The lack of continuous plausible nutrition surveillance data on MNDs makes it difficult to identify and quantify the full extent of the magnitude of the prevalence of MNDs in Ghana.

### 3.3. Nutritional Interventions in Ghana

Ensuring adequate nutrition is a fundamental right of each child; hence, intervening at each point in the human life cycle is crucial to accelerate and consolidate positive change in health outcomes. Well-planned nutrition interventions provide opportunities for addressing the critical factors affecting poverty and can serve as windows of opportunity for consolidating efforts of the education and health sector as well as aid Ghana in attaining the Sustainable Development Goals by 2030. Considering the multifactorial nature of MNDs, this review presents strategies such as nutrition-specific interventions consisting of supplementation strategies, fortification strategies coupled with nutrition-sensitive interventions which include agricultural production and food processing strategies, disease control and WASH strategies and nutrition education, advocacy and economic strategies.

### 3.4. Nutrition-Specific Intervention

The Global Scaling-up Nutrition Network Support describes nutrition-specific interventions as activities pertaining to exclusive breastfeeding for up to 6 months and continued breastfeeding, together with appropriate and nutritious food for up to 2 years, fortification of food programmes and intervention which involve the distribution or administering micronutrient supplements as well as intervention that treat severe malnutrition [49].

Ghana has benefited extensively from several nutrition-specific initiatives; however, there exists very limited plausible evidence of the impact on a national level. The scale and strength of the interventions in terms of statistical significance of findings and level of impact for most of the interventions reported by nutrition-specific interventions remain scant and varied as shown in Tables 6, 7, 8, 9, 10, 11, and 12.

## 4. Supplementation

Supplementation programmes are often characterized by the provision of highly concentrated individual/mixtures of (usually synthetic and lipid-based) vitamins and minerals in the form of powders, capsules, pills or liquids (separately from the diet). Typically, supplements are administered to treat specific MND diseases or to boost the micronutrient profile of vulnerable high-risk groups: infants and young children, adolescent girls and women of childbearing age [97].

In Ghana, the provision of individual or mixture of iron, zinc and folic acid supplementation remains one of the largest and most common programme campaigns in nutrition with limited success rates as shown in Table 6. A 6-month community-based study of 200 infants by [98] sought to determine the effect of vitamin A alone (1,00,000 IU for infants less than 12 months and 2,00,000 IU for children greater) versus vitamin A and zinc supplements (10 mg daily) on the incidence of clinical malaria. The result indicated that supplementation exerted a protective effect against malaria. The number of children who were diagnosed with uncomplicated malaria in the intervention group was 27% significantly lower compared with the children in the control group (*p*=0.03). There were, however, no effects on severe malaria, pneumonia, anaemia and diarrhoea and other anthropometric indices.

Although supplementation intervention provides excellent opportunities for MND treatment on a large scale, efficacy of blanket supplementation programmes is limited [99, 100]. The Obaapa VitA double-blind, cluster-randomized, placebo-controlled trial that assesses the effect of vitamin A supplementation in women of reproductive age (15–45 years) showed that weekly vitamin A supplementation in women of reproductive age has no beneficial or deleterious effect on the causes of infant death at age 6 or 12 months in rural Ghana [101]. The study was conducted in 7 contiguous districts in the Brong Ahafo region of Ghana, live-born infants from 1 June 2003 to 30 September 2008 were followed up through 4-weekly home visits with weekly low-dose (25,000 IU) vitamin A intakes. The results from the 1086 clusters, 62,662 live births, 52,574 infant-years and 3268 deaths yielded 95% cis comparing weekly vitamin A with placebo: 1.04 (0.88–1.05) early infant mortality, 0.99 (0.84–1.18) late infant mortality and 1.03 (0.92–1.16) infection-specific infant mortality. There was no evidence of modification of the effect of vitamin A supplementation on infant mortality by sex (Wald statistic 1⁄40.07, p1⁄40.80) or season (Wald statistic 1⁄40.03, p1⁄40.86).

Subsequently, findings from a study by Edmond et al. [102] also do not support the inclusion of vitamin A supplementation as a child survival strategy in Ghana. The trial studied 22,955 live births between 16 August 2010 and 7 November 2011 with 11,474 randomly assigned to receive vitamin A and 11,481 to receive placebo for 6 months. The study recorded 278 postsupplementation deaths to 6 months of age in the vitamin A group (mortality risk 24·5 in 1000 supplemented infants) and 248 deaths in the placebo group (mortality risk 21·8 per 1000 supplemented infants), relative risk (RR) 1·12 (95% CI 0·95–1·33; *p*=0.183) and risk difference (RD) 2·66 (95% CI–1·25 to 6·57; *p*=0.18). Adverse events within 3 days of supplementation did not differ by trial group. 122 infants died in the first 3 days after supplementation: 70 (0·6%) in the vitamin A and 52 (0·5%) in the placebo group (risk ratio [RR] 1·35, 95% CI 0·94–1·93, *p*=0.102).

Several studies that examined the factors that account for these disparities and the unsatisfactory health outcomes of supplementation among vulnerable groups argue that in contrast to the perception that population-wide supplementation has a direct impact on health improvement, most supplementation campaigns are ineffective due to low compliance rate, poor political commitment/will and financial support, sociocultural and religious differences in beliefs systems of donor and recipient populations [103–105]. Other supplementation programmes also fail to give adequate consideration to confounding factors (hygiene, worms and malaria status) in the planning and implementation of interventions, thereby limiting the individual's response to treatment.

### 4.1. Food Fortification

Food fortification is the addition of macro/micronutrients (natural or synthetic) at near natural levels of nutrients in the naturally occurring food, product or ingredient [106]. This mode of micronutrient delivery is amenable to the delivery of other micronutrients. However, the relative efficacy of multiple micronutrient supplements for the treatment of anaemia requires evaluation due to possible nutrient interactions.

The Ghana Universal salt iodization and iodine deficiency disorder control programme introduced in 1996 remains one the earliest examples of commodity fortification in Ghana. The programme that encompassed two main models was designed to increase the production of adequately iodized salt: the first was local sourcing and distribution of potassium iodate (KIO3), and subsequently, the creation of the Salt Bank Cooperatives (SBC) to allow competitive pricing tailored to local needs of small-scale artisanal salt farmers. Since its initiation, Ghana has recorded an annual reduction in the prevalence of goitre, and coverage of households with adequately iodized salt increased between 1996 and 2006 [56, 57]. Many more recent large fortification programmes including the Ghana Food and Drugs Authority led to mandatory fortification of all commercial vegetable oil and flour for retailers' processors and manufacturers; however, these had limited impact due to challenges in compliance with national standards [3].

Other smaller fortification-based interventions have mainly targeted reducing MND in infants and young children and adolescents (girls of reproductive age) through feeding programme or nutritional intervention. For nearly 40 years prior to the implementation of the National Ghana School Feeding Programme in 2005, the Catholic Relief Services (CRS) of Ghana together with WFP had been running school feeding programmes in the northern regions of Ghana. The mandate was to provide one meal to all children attending catholic primary schools/institutions and two meals for infants in catholic day care facilities nationwide especially in the impoverished northern regions [22]. In the period between 2006 and 2010, its activities intensified with the provision of school-based meals for primary school children (p1–p6) and take-home rations (composed of 150 g of fortified corn soy blend, 3 g of iodized salt and 10 g of palm oil per child) for girls in primary four (P4) to junior high 3, as part of the Ghana Education Service's ‘Education to Benefit Girls' initiative. The take-home rations benefited over 42,000 girls and 25 districts of the three northern regions [107].

A study by [108] looked at the use of home fortification of local Ghanaian weaning foods with ‘Sprinkles' (single-serve sachets containing microencapsulated ferrous fumarate) to treat anaemia. The study examined the relative efficacy of Sprinkles formulated with iron and zinc in anaemic infants, compared with Sprinkles formulated with iron alone through a feeding trial with 304 anaemic infants (mean age 10.3 ± 2.5 months; haemoglobin 87.4 ± 8.4 g/L) in rural Ghana. The results indicated that the rate of recovery from anaemia was higher in the Fe group compared with the FeZn group (74.8 vs. 62.9%; *p*=0.048). These results suggest that this intervention alone was insufficient to improve zinc status or promote catch-up growth in this stunted and wasted population but Sprinkles with iron, or iron and zinc, was very successful in treating anaemia in a controlled setting, home fortification using micronutrient.

Similarly, the KOKOPlus (KP) programme led by the Ajinomoto group aimed at improving the protein intake of young children by fortifying local breakfast meals with macronutrient–micronutrient supplement KP. In an evaluation by [66] the effect of KP provided to infants from 6 to 18 months of age, on linear growth in a single-blind cluster-randomized trial reported that KP was effective in reducing acute infection, improving Hb and demonstrated a dose–response effect on LAZ adjusting consumption. The study evaluated a total of 38 communities randomly allocated to receive KP (14 communities, *n* 322), a micronutrient powder (MN, 13 communities, *n* 329), nutrition education (NE, 11 communities, *n* 319) and a control group (*n* 303). KP supply and morbidity assessment weekly and anthropometry measurement together with NE education were provided monthly. Biochemical indices were measured at baseline, 6, 12 and 18 months. No differences existed in mean LAZ scores at endline (−1.219 (SD 0.06) KP, −1.211 (SD 0.03) MN and −1.266 (SD 0.03) NE). Acute infection prevalence was lower in the KP than NE group (*p*=0.043). Mean serum Hb was higher in KP infants free from acute infection (114.02 (SD 1.87) g/L) than MN (107.8 (SD 2.5) g/L; *p*=0.047) and NE (108.8 (SD 0.99) g/L; *p*=0.051). Compliance was 84.9% (KP) and 87.2% (MN) but delivery 60%. Adjusting for delivery and compliance, the LAZ score at the endpoint was significantly higher in the KP compared to the MN group (+0.2 LAZ; *p*=0.026).

### 4.2. Nutrition-Sensitive Intervention

Reference [109] describes nutrition-sensitive interventions as programmes that tackle the underlying determinants of nutrition such as agriculture, social care/safety nets, early child development and education. The pathways from some of these determinants to nutrition are diverse and often complex but have enormous potential to enhance the scale and effectiveness of nutrition-specific interventions.

The mandate of the current NPP-led Ghanaian government has been stated in the Nation's address [77] and that its focus is to design systems for maximum nutrition and health output from every sector of the economy by making nutrition and health an integral part of government policies and goals.

### 4.3. Agriculture and Food Processing Programmes

Ghana is currently aiming at providing adequate and balanced nutrient output from agricultural activity and food processing programmes not only to attain the SDGs but programmes are being implemented as part of the Ghana Beyond Aid Agenda. The agenda seeks to put in place key strategies for poverty reduction and provide a major source of livelihood for resource-constrained nutritionally vulnerable populations through some of the programmes listed in Table 8.

To address the problem of malnutrition, programmes like the ‘1District 1Factory' and ‘Planting for Food and Jobs and Rearing for Food and Jobs' are tailored towards diversifying agricultural produce through commercialization of agriculture. By so doing, providing the Ghanaian populace with a wide range of cross-seasonal produce, thereby increasing access to food, increasing food security, and encouraging dietary diversification.

Dietary diversification through agriculture diversification empowers individuals and/or populations at large to take charge of their own dietary needs which often translates into informed choice on how to acquire, combine, process (cook) and eat (portion) locally available food [110]. This would substantially reduce the risk of micronutrient malnutrition to the most at-risk people, especially women.

## 5. Advocacy: Nutrition Education—Gender/Social Safety Nets and Economic Strategies

The sustainability of several well-meaning nutrition-specific interventions has been widely attributed to the lack of adequate nutrition education or behaviour change communication [111]. Nutrition education through behaviour change communication in any intervention is crucial for the longevity of impact and effectiveness. MND interventions and prevention that adequately factor in appropriate behaviour change messaging in their planning and implementation are likely to result in long-term sustainable impact and increase the rate of local adaptation and acceptability [112].

Cash transfer for the cultivation of specific functional foods for nutrition such as beetroots and green leafy vegetables helps to build resilience against nutrition metrics that are susceptible to economic transitions. The increased access to finance for farmers through entrepreneurial and market-based programmes like the Ghana Nutrition Links [92]. ENAM project 2009 has been positively associated with food supply diversity (energy from nonstaple crops) in local implementation areas. Investigating the effect of receiving cash transfers with a complementary nutrition-specific intervention (supplementation) showed that the receipt of nutritional supplements in addition to a cash transfer in Niger led to a halving of moderate acute malnutrition relative to receiving the cash transfer alone [113].

In Ghana intervention studies such as [68, 69] and the ENAM project [93], all adopt an integrated approach to nutrition programming. Ghana Health Service [52] indicated that integrated approaches to infant and young child interventions such as exclusive breastfeeding combined with growth monitoring, vaccination and peer counselling continue to increase the uptake of exclusive breastfeeding to the WHO recommendation of 6 months.

Each intervention strategy discussed in the preceding section has its merit and can be applied to various populations, but the underlying principle is that sustainability requires effective implementation guidelines, which are based on evidence-based policies that address dietary deficits peculiar to the population by drawing from the strength of multiple strategies. There is no one-fits-all set of rules for nutritional intervention as the dynamics and determinants vary from population to population even within the same country [74]. This integrated approach helps to identify the unique dynamics (social, cultural, physical and economic) of malnutrition among local populations, control confounders and ensure sustainable nutrition without any drastic change from indigenous food culture [114].

## 6. Environmental, Hygiene and Sanitation

Similar studies integrate environment sanitation, water and hygiene promotion and food safety activities within the target population by incorporating nutrition-sensitive activities such as the WASH initiatives and interventions [115]. Studies have reported that diarrhoeal infestation and parasite infestation among school children adversely affect proxy indicators of nutritional status and are key confounders in the malabsorption of nutrients [69, 93].

## 7. Discussion

The studies included in this review depict a consensus of strategies and frameworks directing Ghana's effort to improve the nutrition among its populace and target MND treatment and prevention. This review also highlights the need to invest in sustainable nutrition strategies as a crucial public health measure to reduce MND disease burden as well as attain Ghana's SDG goal by 2030.

The key observation across the studies shows the use of multiagency efforts as an avenue to control the MND situation in Ghana. It is evident that nutrition-specific interventions like the National supplementation programmes, GHS campaigns on early initiation of breastfeeding, and exclusive and mandatory food fortification programmes have subsequently resulted in Ghana making gradual but steady progress in reducing the prevalence of malnutrition in children under 5 years in the late 2000s [27]. Despite the considerable improvements in the Ghanaian nutrition landscape, vitamin A, iron and zinc deficiencies remain endemic and vital in determining morbidity or mortality [52].

Nutrition-sensitive strategies that address the root causes of MNDs require inputs from various sectors such as agriculture, health, education and social safety nets. For instance, dietary diversification strategies through agriculture diversification like *HortiFRESH* [81, 82] and *Rearing for Food and Jobs* [77] empower individuals and/or populations to take charge of their own dietary needs which often translates into informed choice on how to acquire, combine, process (cook) and eat (portion) locally available food [110]. This is likely to substantially reduce the risk of micronutrient malnutrition to the most at-risk people, especially women.

The complexities of simultaneously planning for future MND risk and managing the current MNDs attributable burden on the Ghanaian public health sector is taking its tow on its already limited resources [27]. Considering the current strategic direction of Ghana's development efforts, improving the health of its populace and attaining the SDGs will require enormous investment in sustainable agriculture, nutrition and health. Addressing MNDs in Ghana requires a concerted effort that transcends the traditional disciplinary boundaries of health and nutrition programming like providing nutrients or fortified nutrient-dense food varieties, as MNDs are rarely caused by a single factor and usually stem from a combination of dietary, environmental, economic and social factors.

Effective MND prevention and treatment strategies will require greater consideration of integrated strategies like those described in the studies by Gelli et al. [68], Azagba-Nyako [69] and the ENAM project [93]. Ghana Health Service [52] indicates that integrated approaches to infant and young child MND interventions such as exclusive breastfeeding combined with growth monitoring, vaccination and peer counselling continue to increase the uptake of exclusive breastfeeding to the WHO recommendation of 6 months which helps to ensure optimal child growth and development.

While these interventions are ineffective against acute MNDs, they should be complemented by broader efforts aimed at promoting dietary diversity, enhancing agricultural practices, strengthening healthcare systems, improving sanitation and hygiene practices and empowering communities to address the multifaceted nature of the MNDs. Furthermore, other research studies can aim at incorporating foods such as sweet potato and orange flesh potatoes into a complementary food mix while assessing their acceptability, nutritional content and efficacy in reducing MNDs. This is mainly to reduce the phytate content of complementary food-mix which is influenced by some cereal ingredients [116]. Also, strong partner relations with key stakeholders go a long way to ensure success in nutrition programmes [117]. Thus, further studies can be rolled out to assess the impact of stakeholders in effective nutrition programmes. Each intervention strategy discussed in the preceding section has its merit and can be applied to various populations.

## 8. Conclusion

MNDs in Ghana need multisectoral interventions between nutrition-specific and nutrition-sensitive interventions. Although some of the interventions, such as the National Food Fortification Programme and the School Feeding Programmes, have made quite impressive progress, their eventual sustainability depends on the development of efficient implementation guides and evidence-informed policies to address specific dietary needs.

The multiagency collaboration has proved useful, with successful partnerships so far forged between government agencies, NGOs and international organizations. So far, the partnerships formed have advanced holistic approaches to addressing the root causes of the MNDs through dietary diversification, with agricultural initiatives like HortiFRESH and Planting for Food and Jobs, nutrition education remaining key and disease control measures.

However, the review also indicates large gaps, particularly in continuous nutrition surveillance information and improvement of policy implementation. The need, thus, to address such gaps becomes relevant for sustainability in the effectiveness of the MND interventions. Some of the new areas of inquiry suggested in further studies are the inclusion of nutrient-dense foods, such as sweet potatoes, into the complementary food blends and establishment of stakeholder roles among others for the effective delivery of the nutrition programmes. Ultimately, the MND interventions are to be taken to scale in Ghana and must be adapted to various socioeconomic contexts of the local populations, ensuring nutrition is sustainable without upsetting the balance of the indigenous food cultures. Drawing on a number of approaches and building strong partnerships, Ghana is therefore likely to continue the reduction in MNDs in her contribution towards the attainment of her Sustainable Development Goals by 2030.

## Figures and Tables

**Table 1:** Selection criteria model for the review.

Framework stage	Description
Identify the research questions	• Identifying the research questions provides the roadmap for subsequent stages. Relevant aspects of the question must be clearly defined as they have ramifications for search strategies. Research questions are broad as they seek to provide limits to the study.

Identify relevant studies	• Identifying relevant electronic databases, reference lists and hand searching of key journals organizations and conferences. • Identifying the relevant studies and developing a decision plan for where to search, which terms to use, which sources are to be searched, period and language.

Study selection	• Using keywords, post hoc inclusion and exclusion criteria for study selection

Collate, summarize and chart the data	• Tabulation extracts data from each study under each key stage

Results reporting AND knowledge synthesis	• An analytic framework or thematic construction is used to provide an overview of the breadth of the literature but not a synthesis. A numerical analysis of the extent and nature of studies using tables and charts is presented. A thematic analysis is then presented. • Fill the data gap. Provide opportunities for stakeholder involvement to suggest additional questions and pathways and provide insights beyond those in the literature.

**Table 2:** Summary of the identified research questions.

Phase	Key questions
Scoping review exercise	• What are the micronutrient deficiencies in Ghana? • What are the key national policies and frameworks targeting MNDs in Ghana? • Who are the key actors and agencies implementing programmes in micronutrient deficiency prevention and what do they do?
	• What are the core nutrition-specific strategies utilized in micronutrient deficiency intervention in Ghana? • What are the food-based strategies and how are they being implemented? • What are the nutrition-sensitive interventions mainstreaming micronutrient deficiency prevention?

Synthesis of data	• What are the existing synergies, barriers and limitations of the current strategies? • What are the strategies to optimize the existing synergies to overcome barriers and limitations in micronutrient intervention programmes? • What additional research is needed?

**Table 3:** Number of studies published on micronutrient-related interventions between 2000 and 2023.

Item	Quantity
Eligible number of studies	46
Studies on nutrition-specific strategies	31
Studies on nutrition-sensitive strategies	15
National supplementation strategies	5
Fortification strategies (use biofortified crops, fortified foods, flour, oil or micronutrient powders for home fortification)	14
Disease control strategies	7
Dietary diversification strategies	5

**Table 4:** Summary of national nutrition-related legal framework and policies.

Document/policy/act	Date/implementing agency/partners	Aim	Reference
Ghana health policy: Creating Wealth through Health	• September 2007 • Ministry of Health	• Redirecting health interventions towards health promotion • Preventive health with emphasis on nutrition and rehabilitation	[16]
Exclusive breastfeeding	• May 2000 • Ghana Health Service • FDA regulation PNDCL305B	• Funding to strengthen • Promote and support the practice	[17]
Ghana nutrition policy	• March 2013 • Ministry of Health • Ministry of Food and Agriculture • Ministry of Water Resources, Works and Housing • Ministry of Environment, Science and Technology • Ministry of Education • Ministry of Trade and Industry Minister for Finance • Ministry of Local Government and Rural Development • Ministry of Gender, Children and Social Protection • National Development Planning Commission	• Providing a framework for relevant ministries to align their policies and programmes to contribute to a reduction in undernutrition. • Guide the process of prioritizing nutrition challenges for action. • Providing a basis for selecting and implementing priority. • Providing strategies for prevention and control of malnutrition. • Facilitate mobilization of resources for nutrition programming across all relevant sectors and institutions.	[18]
School health policy	• 2014 • Ghana Education Service • Ministry of Education	• Providing nutrition education in schools. • Enforcing water, sanitation and hygiene power CFG/battery (WASH) goals at school level.	[19]
Education strategic plan	• 2010–2030 • Ministry of Education	• Expand and improve school health, sanitation and safety systems. • 100% of basic education schools must have hygiene systems and sanitation by 2015. • 75% of basic education schools will have access to potable water by 2015.	[20, 21]
Health sector gender policy	• April 2009 • Ministry of Health • Ministry of Women and Children's Affairs	• Mainstreaming gender in the planning • Implementation of health sector programmes.	[22]
Community-based Health Planning and Services Policy Accelerating Attainment of Universal Health Coverage	• November 2014 • Ghana Health Service • Municipal and district assemblies	• Providing nutrition education and support and growth monitoring and promotion. • Improving community-integrated management of childhood illnesses.	[23]

**Table 5:** Adjunct government agencies affiliated with the nutrition agenda in Ghana.

Agency	Responsibility/core activities	Reference
Ministry of Food and Agriculture (MoFA)	• Plan and advise the government on agricultural development policies, administration and management of the agricultural sector • Monitor and evaluate the agricultural sector on development in crops, livestock, irrigation and mechanization • Develop agricultural programmes and projects	[24]
Women in Agriculture Development Directorate (WIADD)—Ministry of Food and Agriculture	• Develop intra- and intersectoral collaboration for the development of policies, programmes and projects that enhance the livelihoods of women farmers and processors. • Liaise with research and extension to analyse women-specific challenges in the agriculture sector and seeking solutions.	[24]
Ghana Education Service	Running school-based health education programmes.	[25]
Department for Community Development—Ministry of Local Government	Promotion of the development of vulnerable groups in the rural and urban areas with focus on women and children.	[8, 22]
Ministry of Gender, Children and Social Protection Affairs	Running the Ghana School Feeding Programme.	[26]
Department of Social Welfare—Ministry of Employment	Promotion of rural development issues, including the welfare and development of vulnerable groups in the rural and urban areas with focus on women and children.	[26–28]
Ghana Statistical Service	Collection of nutrition surveillance through GDHS surveys	[8, 29, 30]
Council for Scientific and Industrial Research—Food Research Institute	• Conduct applied market-oriented research on food processing and preservation, food safety, storage and utilization, and national food and nutrition security. • Value addition to food commodities in the country and nutritional composition (proximate, minerals, vitamins and antioxidants) analysis.	[31]
The Council for Scientific and Industrial Research-Crops Research Institute	Developing improved crop varieties that are drought resistant and deliver specific micronutrients.	[32]
Department of Community Nutrition, University of Development Studies	Delivery of community nutrition.	[33]
Food Science and Nutrition Department, University of Ghana	Education and research on food science and nutrition.	[34]
Food Science and Technology Department, Kwame Nkrumah University of Science and Technology	Postharvest and food processing technology, storage systems and methods to maintain the qualities of the focused food commodities to prevent postharvest losses.	[35]
Nutrition Department, Noguchi Memorial Institute for Medical Research (NMIMR)	Running infant and young child nutrition, food consumption and food security interventions.	[36]
School of Public Health, University of Ghana	Research on public nutrition and food science, collecting nutritional surveillance data.	[37]

**Table 6:** Nongovernmental organizations involved in nutrition-related interventions.

Agency	Core activities in Ghana	Reference
World Food Programme (WFP)	Food security, food assistance, nutrition safety nets, poverty reduction or income support goals through cash transfers.	[39]
Food and Agriculture Organization (FAO)	Critical support for developing safety net systems for food security, national nutrition programme, assessment monitoring and vulnerability mapping activities.	[40, 41]
Partnership for Child Development (PCD)	Home-grown school feeding systems.	[42]
World Health Organization (WHO)	Critical support for developing healthy systems.	[43]
United Nations Children's Fund (UNICEF)	Nutrition education and supplementation programmes	[4]
Netherlands Development Programme (SNV)	Nutrition sensitivity agriculture, strengthen agricultural, environmental systems for health.	[41]
Japan International Cooperation Agency (JICA)	Capacity building for local food production and nutrition intervention programmes.	[44]
Catholic Relief Services (CRS)	Feeding intervention for infants' young children and adolescents.	[45]
CARE International	Education, support farmer access to markets	[46]
International Food Policy Research Institute (IFPRI)	Food and nutrition policy drafting, monitoring and evaluation.	[47]
Alliance for a Green Revolution in Africa (AGRA)	Agricultural systems through direct collaboration with local farmers.	[48]

**Table 7:** Summary of supplementation interventions in Ghana.

Programme/project	Population	Target nutrient	Duration	Implementing agencies	Target food	Coverage level	Efficacy	Reference
Iron and Folate Supplementation (IFA) (SPRING) project	Mothers with children 0–5 months 90 or more days during pregnancy	(30–60 mg iron, 0.4 g folic acid)	2006–present	Ghana Health Service	Oral administering	National	Approximately 59.4%	[50]
Vitamin A Supplementation (VAS)	Children 12–59 months	200,000 IU VAS (1,00,000, 6–11 months of age)	1991–2000	Ghana Health Service	Oral administering	RCT *n* = 5066 Navrongo, a rural area of northern Ghana	22.0% of children 12–59 months received vitamin A supplementation only 19.9% received two doses of VAS in a calendar year	[51]
GIFTS: Girls' Iron-Folate Tablet Supplementation	School adolescent girls and out-of-school adolescent girls aged 10–19 years	60 mg of elemental iron and 400 μg (0.4 mg) Folic Acid] once every Wednesday un	2016–2019	Ghana Health Service UNICEF, WHO Canadian government, CDC), USAID Korea international cooperation Agency (KOICA) GES.	Oral administering	National In school In health facilities	3,60,000 in-school adolescent girls and close to 6,00,000 out of school girls, in four regions.	[52]
Maternal and Infant Lipid-based Nutritional Supplementation International Lipid-based Nutrient Supplements (iLiNS)-DYAD trial in Ghana	Women at ≤ 20 weeks of gestation	Iron and folic acid 200 mg calcium/d for 6 months postpartum Multiple micronutrients (1-2 RDA of 18 vitamins and minerals) Lipid-based nutrient supplements (iLiNs)-DYA daily 20 g (118 kcal) lipid-based nutrient supplements (LNS)	2009–2014	University of California, Davis from the Bill & Melinda Gates Foundation		RCT with *n* = 1320 Yilo and lower Manya Krobo districts of the eastern region of Ghana between December	LNS on height at 4–6 years in this cohort, which had a low stunting rate, but height was greater in the LNS group among children of non-overweight/obese women.	[53]
Vitamin K administration	Newborns 60 min after childbirth	Vitamin K	2011	Ghana Health Service UNICEF USAID	Oral administer	Nationwide 10 regions of Ghana	Approximately 45.9% of newborns	[54]

**Table 8:** Summary of fortification interventions in Ghana.

Programme/project	Target population	Target nutrient	Duration	Implementing agencies	Target food	Coverage	Efficacy	Reference
Iron fortification	Adolescents and women 20 years and above	Iron		GHS, UNICEF	Wheat flour	Nationwide	TBC	[55]
National food fortification programme	All age groups	Vitamin A	2007–present	Food and Drugs Authority and Health Service (GHS), the Food and Drugs Board (FDB) and the Ghana Standard Board (GSB) are funded by the Global Alliance for Improved Nutrition (GAIN)	Vegetable oil	National, mandatory	42% in 2009. The percentage of adequately fortified vegetable oil increased from 69% to 95% between 2010 and 2011.	[56]
National wheat fortification	All age groups	Vitamin A	2007–present	Ghana Standards Authority Food and Drugs Authority	Wheat flour	National, mandatory	No date of implementation coverage due to issue with adverse effects on flour taste	[56]
Universal salt iodization and increased iodine deficiency disorders control programme	Adolescents Infants/young children Lactating w/pregnant women Women of reproductive	Iodine	1996–present	GHS University of Ghana UNICEF Food and Drug Authority (FDA)	Salt	Nationwide 10 regions of Ghana	29.3% of households were using adequately iodized salt.	[57]
Premix and iron fortification	Children (6–35 months)	Vitamin A, vitamin C iron	5 months	GHS, FDA, Kintampo Health Research Centre, hospital for sick children	Semisolid foods (cereal mix)	Bono region	Continuous inclusion of micronutrient powder containing prophylactic iron decreased anaemia, iron deficiency and iron deficiency anaemia among children	[58]
Sprinkles, crushable Nutritabs	Infants	Fat-based Nutributter vitamins and minerals	Feb–Sep 2004	University of Ghana	Fermented cereal porridge and others	105 infants	Increased ferritin concentration	[59, 60]
Sprinkles	Infants	Iron Zinc	2000–2003	UNICEF	Maize-based porridge, infant meal	304 infants	Decreased rate of anaemia, 74.8%	[61, 62]
Micronutrient sprinkles	Children (16–18 months)	Iron	8 weeks		Maize-based porridge probably	133 children	Decrease in iron deficiency anaemia	[63]
Premix	Women and children (1–5 years)	Iron and iodine	1997–1998	Micronutrient initiative Canada	Salt	184 women, 82 children Sekyere west	Decreased level of anaemia in both women and children	[64]
NaFeEDTA	School-aged children	Iron	7 months	University of Development Studies (Ghana)	Whole cowpea meal	241 SAC	47% reduction in Fe deficiency	[64]
Sprinkles	Infants (7–9 months)	Vitamin A	January through June 2010		Porridge (not specific)	870 infants	4·9% of the children were diagnosed with VA deficiency	[65]
*KOKOPlus*	IYCF	Micronutrient powder	2012–2014	USAID, JICA and Ajinomoto	Soybean	1273 infants from 6 to 18 months of age	*KOKOPlus* reduced acute infection and improved Hb dose–response effect on length-for-age z-score adjusting consumption for delivery.	[66]

Abbreviation: TBC = total blood cell.

**Table 9:** Summary of dietary diversification interventions in Ghana.

Programme/project	Target population	Target nutrient	Duration	Implementing agencies	Target food	Coverage	Efficacy	Reference
Ghana School Feeding Programme	School-aged children in selected schools nationwide	Dietary diversification	2005–present	Government of Ghana	One hot meal a day	1.69M children, 37.4% of national coverage	Improve nutrition and school attendance	[26, 67]
Home-Grown School Feeding Programme	School-aged children (aged 5–15 Y).	Dietary diversification Micronutrient powders	2013–2016	Partnership for Child Development	School meals	2869 school-aged children		[68]
School meal	School-aged children (aged 5–15 Y	Dietary diversification Food to food fortification	2014–2016	University of Westminster GES lower Manya Krobo	Indigenous recipes	330 SAC	Treatment group had an average 3.24% increase in height (*p* ≤ 0.05) and 13.08% increase in weight (*p* ≤ 0.0.5)	[69]
Dietary diversity and haemoglobin	Women of reproductive age (WRA) (15–49 years)	Iron	2018	University of Ghana	Common foods and indigenous recipes	153 WRA in the Binduri district, of the Upper East region	Dietary diversity score was a strong predictor of haemoglobin concentration	[70]

**Table 10:** Summary of disease control strategies.

Programme/project	Target population	Input	Duration	Implementing agency	Coverage	Efficacy	Reference document
Early breastfeeding initiation (EBF)	Postpartum women	Service monitoring evaluation	2016	Ghana Health Service	National	64,651 (98.4%) lactating mothers initiating EBF within 30 min of birth Only 56.6% of mothers of children 0–2 years who gave birth in a health facility breastfed their infants within 1 h of birth	[55]
Exclusive breastfeeding	Postpartum women with infants	Service monitoring evaluation	2014-present	Ghana Health Service	National	64,651 infants 98.4% national coverage 52% of infants are exclusively breastfed during 6 months after birth (	[55]
Ghana Child Growth Monitoring	WRA with children under 5 years	Monthly health promotion/education facility/outreach contacts	2016	Ghana Health Service	National	100,944 out an expected number of 105,963 children within the ages of 0–11 months representing 95.3% were registered during growth monitoring and promotion in	[52]
National deworming exercise coverage	SAC In KG-JHS	500 mg albendazole or Mebendazole	2016–2020	Neglected tropical diseases. Programme (NTDP) GHS & GES	6 million SACS three in 205 districts	Selected regions	[71]
Mother and baby-friendly Health Facility Initiative (MBFHI)	Pregnant women and postpartum women	Staff training	2013–present	Ghana Health Service Determine the effect of hospital status of baby-friendly hospitals (BFI) and non–baby-friendly hospitals (NBFH) on infant feeding practices and infant growth	150 pregnant women and postpartum women	Initiation of BF: Relatively good knowledge BFH and NBFH = 90% (*p*=0.012), support of bf position: NBFH had more knowledge than BFH (*p*=0.016), women from NBFH were giving prelacteal feeding more than women from BFH (*p*=0.37)	[52]
Ghana Nutrition and Malaria Control for Child Survival Project (NMCCSP)	0–24 months children pregnant women	1,550,000.00 long-lasting insecticide-treated nets	2006–2013	MOH	400,000.00 65,000 children under 2 years	77 in the Northern, Upper East, Upper west, Central, Volta, Ashanti and Greater	[29]
Ghana Treatment and Management—Community-based management of acute malnutrition (CMAM)	Initial utilization—children 6–59 months with severe acute malnutrition (SAM)	Supplementary feeding F75, F100 and RUTF	2010–2013	Ghana Health Service UNICEF	82% coverage for outpatient therapeutic care (512 of 624 health centres).	73% cure rate Only 32.5% of children 6–59 months with severe acute malnutrition (SAM) admitted for treatment at health facilities	[72]

**Table 11:** Summary of agriculture and food processing programmes.

Intervention	Project/implementing agent	Location	Year	Coverage/target population	Summary of findings	Reference
Boosting Green Employment and Enterprise Opportunities in Ghana	SNV European Union, the Embassy of the Kingdom of the Netherlands in Ghana, the United Nations Capital Development Fund (UNCDF) and SNV	Ashanti and western regions of Ghana.	2015	Small and medium enterprises (SMEs), offering decent and sustainable jobs to youth, women	• At least 5000 people trained and coached for employability and entrepreneurship (youth: 60%–80%, women: 40%–60% and returnees: 10%); • At least 3500 people have created or developed self-employment opportunities (youth: 60%–80%, women: 40%–60% and returnees: 5%–10%); • At least 100 MSMEs have been incubated or accelerated to expand their business (disaggregated by number of employees, sector and district); • At least 1500 decent and sustainable jobs created by MSMEs (youth: 60%–80%, women: 40%–60%)	[73, 74]
2SCALE the largest incubator for inclusive agribusiness in Africa	IFDC, BoPInc and SNV. The programme is funded by the Netherlands Ministry of Foreign Affairs	Nationwide	2012–2020	Northern regions of Ghana Incubating inclusive Agribusiness entrepreneurial producer organizations Local SMEs that trade or process local farmers	• Scale over 60 public–private partnerships • Improve the livelihoods of over 750,000 farmers (50% women, 40% youth) • Develop new business for more than 5000 SME and producer organizations (2500 female-headed, 1000 young entrepreneurs) • Improve access to nutritious food for at least 1,000,000 BoP consumers • 375,000 ha covered by eco-efficient agricultural practice • 20,000 new nonfarming jobs (10,000 for women, 8000 for youth) • €50,000,000 coinvestment from the private sector	[75]
Food Procurement Governance for Home-Grown School Feeding	Partnership for Child Development, SNV Ghana School Feeding Programme	Nationwide	2017–present	Developing a more inclusive and responsive relationship between smallholder farmers and school feeding buyers in Ghana	• Improved public procurement policy, regulation and implementation to become inclusive to smallholder farmers. • Enhanced supply chain governance to prepare farmer organizations to supply to school feeding programs and other formal markets. • Institutionalized social accountability processes to facilitate the linkage between smallholder farmers and school feeding.	[41]
Planting for food and jobs	Nutrition Links project	National	2014	Vulnerable groups (women, children)	Enhanced access to food crops all year round to ensure better health and nutrition outcomes	[76, 77]
Rearing for Food and Jobs	ENAM project	National	2004–2009	Women and infants	• Increased consumption of animal-source foods • Reduced risk of child growth faltering	[76, 77]
Commercial Agriculture project for Ghana	Ministry of Food and Agriculture United States Agency for International Development (USAID)	Kpong and Tono	2013–present	Farmers and investors	• Land development, to access to farm road, warehouse construction	[78, 79]
Partnership for Productivity, Protection and Resilience in Cocoa Landscapes (PPPRCL)	DFID and implemented by a consortium with SNV, the nature conservation research centre (NCRC), agro-eco, COCOBOD, the forestry commission and Touton S.A. (lead implementer).	National	2017–present	Farm land Farmers	• Developed and piloted community-based governance frameworks to protect forests; • Developed and strengthened market-based services to increase farmers' productivity in an environmentally sustainable manner; • It has also become possible to easily monitor renovation and rehabilitation (R&R) actions in cocoa landscapes.	[80]
HortiFRESH	Resilience B.V., Advance Consulting, SENSE and Wageningen University and research CDI embassy of the kingdom of the Netherlands in Ghana.	National	2017–2021	• Linking vegetable producers to more efficient markets, including other value chain operators	• Reached 15,000 farmers and hoped to increase their productivity by 20% until 2021. • Improving the business climate and further professionalizing the value chain for vegetable production and consumption in Africa	[81, 82]
Ghana grain development project	Government of Ghana, CIDA	National	2005	Farmers Farming communities	• Development of improved varieties of maize and legumes	[83, 84]
Root and Tuber Crops Improvement Project (RTIP)	International Institute for Tropical Agriculture (IITA)	National	2003–present	Farming communities	• 7,20,000 beneficiaries reached; • Calorie consumption during lean season in beneficiary households increased by 20%; incomes in beneficiary households increased by 15%. RTIP records show it having reached 120,000 households to date	[85]
WAAP (improving roots and tubers)	West and Central African Council for African Research and Development (CORAF/WECARD) Ministry of Food and Agriculture	National	2008–present	Farmers	• Generation and dissemination of technologies to improve roots and tuber production	[86, 87]
Pineapple production on large scale	Ghana investment promotion centre, Ghana free zones board	Accra, Aburi-Nsawam, Awutu	2015	Accra, Aburi, Nsawam, Awutu	• Increased production of pineapples for domestic sale and export • Increase the availability of fruits for local consumption	[88]
Soybean production on a commercial scale	Ghana Investment Promotion Centre	Brong Ahafo and Northern Ghana	2015	Brong Ahafo, northern Ghana	• Increased production and processing of soybean for commercial purposes	[89]
IFAD Agricultural value chain investment	International Fund for Agricultural Development	Rural areas	2014–present	Rural areas in northern Ghana to establish	• 4000 smallholders with improved water management • Maize yield from 1 to 3 tons per hectare, and in soya yield from 0.7 to 2 tons per hectare • 15,000 people trained in climate change resilience actions. Improved agriculture, improved livelihood of rural folks, increased market avenue for rural agriculture	[90]
Sustainable agriculture for industrialization and economy growth	FAO Ministries of Food and Agriculture, health, finance and economic planning, land and natural resources, environment, science, technology, local government and rural development	Rural areas	2018–2022	Rural areas	• Increased food security and nutrition, engaging more women in value-added activities	[40]

**Table 12:** Summary of nutrition-sensitive strategies implemented in Ghana.

Project/programme name	Target population	Implementing agency	Date	Input	Coverage	Ref and date
GoodLife, Live It Well (nutrition and health)	National adolescents Adult men and women Lactating women Pregnant women	WHO, Ghana media	2016–2017	Awareness campaign on how to consume healthier diets, fruits and vegetables in the diet.	National	[91]
Home-Grown School Feeding	School-aged children (SAC)	Partnership for Child Development	2013–2016	Menu planning guide	National	[68]
WASH in Schools (WinS)	School-aged children (SAC) kindergarten/school	Ministry of Education Ghana Education Service UNICEF	2012–present Mandatory	WASH facilities	National School Health Education programme unit	[19]
Nutrition Links project	Ghana national	University of Ghana, World Vision McGill University	2013–2018	Intensive nutrition, health and agricultural training along with provision of home gardens and animal husbandry	705 adolescents (9–12 years)	[92]
Nutrition Links project	181 children under the age of 5 years	World Vision McGill University University of Ghana	2013–2014	Nutrition education enhances access and utilization of animal-source food among vulnerable families with children under 5 years	227 children under 5 Three ecological zones (northern belt, middle belt, southern belt) of Ghana	[92, 93]
Nutrition Links project	181 children under 5	World Vision McGill University University of Ghana	2013–2014	Nutrition education enhances access and utilization of animal-source food among vulnerable families with children under 5 years	227 children under 5 Three ecological zones (Guinea savannah, forest-savannah transitional, coastal savannah)	[92, 93]
National improve Health and well-being through Media	General populace	Range of mass media and social communication for health	2016	Advocacy Behaviour change campaign	Repositioning and repopulating GHS/HPD flagship health promotion brand GoodLife, Live it Well, collaboration and coordination with partners	[94]
Nutrition education intervention on nutritional status of undernourished children	*N* = 153 aged 6–24 months	Determine the impact of nutrition education intervention on the nutrition status of under nourished children.	2018	Randomized cluster	Improvement in underweight, wasting, mica at postintervention differed significantly for underweight (*p*=0.001), wasting (*p*=0.001) and MUAC (*p*=0.001) compared to preintervention.	[95]
ENAM project	Children 2–5 years.		(2004–2009)	Quasi‐experimental longitudinal design addresses caregivers' income and knowledge barriers to optimal child feeding with emphasis on increasing access to animal source foods (ASF) through integrated microcredit, entrepreneurship training, and nutrition education	Free ecological zones in Ghana specifically, the Guinea savannah, forest–savannah transitional and coastal savannah zones	[96]

## Data Availability

The authors confirm that the data supporting the findings of this study are available within the article.

## Nomenclature

AOAC: Association of Analytical Communities
CSO: Civil society organizations
GAIN: Global Alliance for Improved Nutrition
GDHS: Ghana Demographic and Health Survey
GDP: Gross domestic product
GES: Ghana Education Service
GHS: Ghana Health Services
GSS: Ghana Statistical Services
GOG: Government of Ghana
MDG: Millennium Development Goals
NEPAD: New Partnership for African Development
NGOs: Nongovernmental organizations
SDG: Sustainable Development Goals
SEND: Ghana—Social Enterprise Development Foundation of West Africa
SFP: School Feeding Programme
SIGN: School Feeding Initiative Ghana Netherlands
SNV: Netherlands Development Organization
WASH: Water, sanitation and hygiene
WHO: World Health Organization
PICO: Participants, intervention, comparison and outcome
RCT: Randomized controlled trial
RR: Relative risk
RUTF: Ready-to-use therapeutic food
SAM: Severe acute malnutrition
WFP: World Food Programme
